# Older migrants’ perceptions of places to meet: Insights for social work practice

**DOI:** 10.1371/journal.pone.0292730

**Published:** 2023-11-28

**Authors:** Hanna Mac Innes, Anna Dunér, Susanne Gustafsson, Lisbet Lindahl

**Affiliations:** 1 Department of Social Work, University of Gothenburg, Gothenburg, Sweden; 2 Department of Rehabilitation, Jönköping University, Jönköping, Sweden; 3 Department of Research and Development (FoU i Väst), The Gothenburg Region Association of Local Authorities (GR), Gothenburg, Sweden; Walden University, UNITED STATES

## Abstract

This study aimed to explore the experiences of older migrants’ (70+) access to and participation in different meeting places. Qualitative interviews were conducted with participants originating from Finland and four countries in the Western Balkans: Bosnia- Herzegovina, Croatia, Montenegro, and Serbia. The participants used everyday places in the neighborhood, which were not primarily meant to be meeting places, to create and uphold social contacts. These meeting places contributed to experiences of community and trust. Both everyday meeting places and organized meeting places were used to establish and develop relationships that could result in an exchange of both practical and emotional support. Perceptions of “not belonging”, limited proficiency in Swedish, and a strained financial situation created barriers to accessing some meeting places. The results of this study demonstrate the significance of meeting places that are not purposefully aimed at older people in general or older people from a specific country.

## Introduction

This article focuses on older migrants in Sweden and their experiences of access to and participation in different meeting places. In Sweden, twelve percent of people aged 65 years and older have migrated to Sweden at some point during their lives, and this amount is presumed to double in size by the year 2030 [1]. However, older migrants are a heterogeneous group in terms of country of origin, reasons for migration, age of migration which may result in differences in health, living conditions, and degree of social inclusion [2]. Migrants in Sweden are more frequently exposed to social exclusion, entailing income poverty but also the risk of being socially isolated [3]. Other barriers to form and maintain social contacts outside of the family context after migration may be a shorter residence in the new country and restricted skills in the language spoken in the new country [4].Promoting the access to meeting places for older migrants, who are at risk of social isolation [5], may be a way to promote social connectedness among this group and reduce the risk of poor health; risk of depression, cognitive decline and mortality [6]. Since older migrants in Sweden face a higher risk of being poor in older age [7]. The financial aspect of accessibility to meeting places may therefore be argued for this group. For example, correlations between low income and low education and high use of library as a meeting place have been found in studies from Norway. More knowledge is needed regarding how older migrants experience and value meeting places. Gaining more insight into this matter may entail a way of finding health promoting interventions for his group.

Various definitions exist of what constitutes a meeting place. It can be defined as a place for social gatherings encompassing organized social venues such as associations, interest organizations, or senior centers arranged by Long-term care services. Furthermore, it can relate to spontaneous meeting places such as cafés, squares, and parks. Even though the physical environmental aspect of a meeting place is crucial, it can also be viewed as a social construction, as individuals create the meaning of a meeting place in their social interaction with others [8]. Previous research on meeting places for older people has often focused on senior centers, also known as Community Centers for seniors. Extensive research from the North American context describes this type of meeting place ([9–12]). This research indicates that older migrants are less likely to participate in senior centers and points to the need for such places to adapt to the changing interests and needs of the older population ([5, 10, 11]). Since the risk of being poor is higher among older migrants, some meeting places may be more accessible for some groups of older migrants in the older population.

Furthermore, negative perceptions of ageing can create barriers to participation in social meeting places explicitly aimed at older people ([5, 10]). Most studies conducted in the Swedish context on older migrants’ participation in meeting places have focused on senior centres targeting older people from a specific country [13–15]. The results from these studies show that such meeting places are regarded as opportunities to meet people who speak the native language but that such places create a dilemma since they, at the same time, can be encountered as barriers to social inclusion. There is a lack of studies that have applied a more open definition of what constitutes a meeting place and which meeting places older migrants consider valuable in establishing and maintaining social contacts.

For gerontological social work, it is essential to gain more profound knowledge about older migrants’ experiences of the significance of meeting places to promote social-inclusion. The knowledge sought is especially valuable to live up to the ethical values of social work, promote participation in society, and secure equal opportunities for all to access health and social care services. Furthermore, as older migrants are at risk of poorer health and since meeting places are essential to the wellbeing, the topic of older migrants experiences of meeting places can also be motivated from a health and wellbeing perspective ([5, 16]). Increased health risks emerge among people migrating late in life and those who have been in the country for more extended periods ([17, 18]). In summary, while literature focusing on meeting places targeting older people in general or older people from a specific country are vast; less attention has been given to which meeting places older migrants consider as valuable in establishing and maintaining social contacts. The aim of this study was, therefore, to explore the experiences of older migrants’ access to and participation in different meeting places.

## Methodology

### Study design and setting

The study was implemented in a district of a larger city in Sweden with a population of approximately 500,000, of whom 50,000 live in this district. The town is highly segregated, and the longevity between the wealthiest and most impoverished neighborhoods differs by about nine years. The inhabitants in the area come from over 100 countries. Half of all inhabitants in the urban community were born in non-Nordic and outer European countries. Regardless of the person’s age, the most common country of birth was in descending order: Iraq, Iran, Finland, Bosnia-Herzegovina, and the former Yugoslavia. The latter three were dominant countries of birth for persons 65 years or older. In the urban district 2012, residents’ general educational level and income level were lower, and the sickness rate was higher than in the rest of the city’s population. This qualitative interview study is nested within a more extensive health-promotion study:" Promoting Ageing Migrants ‘Capabilities” [19]. The target group consists of people from two of the largest migrant groups in Sweden, Finland, and our countries in the Western Balkans: Bosnia-Herzegovina, Croatia, Montenegro, and Serbia. See the protocol for detailed information on the more extensive health promotion study [19]. One hundred thirty-one people, 70 or older, participated in the health-promotion study. The inclusion criteria were born in Finland or one of the four countries in the Western Balkans. In addition to this selection criteria were a person living in ordinary housing in the urban district and not dependent on informal or formal help with daily activities. The single exclusion criterion was impaired cognition.

### Participants

For the present study, a strategic sample of ten participants reflecting heterogeneous migration backgrounds were drawn from the larger study. The only exclusion criterion is impaired cognition (Mini-Mental State Examination (MMSE) below 80% of administered items. Five of the participants originated from the Western Balkans and five from Finland; half of them were men, and half of them were women. Five of the participants had migrated as labour migrants, four of the participants as refugees, and one participant for family reasons. Half of the participants had started a university or college education. Most participants had lived in Sweden for 21 years and rated their health good. Less than half of the participants were living alone. The ages of the participants ranged from 70 to 80 years. Data on the income level of the participants was not retrieved. However, half of the group had arrived in younger years as labour migrants, implying a more extended period in the labour market to accumulate enough years and pension rights to obtain a full base pension. The rest of the group had arrived during the financial crisis in Sweden during the 90´s [19]. Most participants arriving later in life suffered financial constraints due to short or no previous history in the labour market.

### Data collection and analysis

Based on a thematic interview guide [20] qualitative semi-structured interviews were performed in 2014. The interview guide focused on experiences of access and participation in social meeting places. The questions in the semi-structured interview guide aimed to attain an exploratory approach to detect the different meanings that participants assigned meeting places. Due to the exploratory approach, [MOU1], we wanted to leave the definition of meeting places (places to meet) as open as possible. The interview guide comprised some introductory questions on general background information about the person’s name, age, years of residence in Sweden, reason for moving to Sweden and current and previous employment. Secondly, interviewees were asked how they perceived their opportunities for social contacts in their current situation. Questions about meeting places were asked. What kind of meeting places they attended, and the significance of these meeting places concerning each other. There were follow-up questions regarding perceived opportunities for social support, giving and receiving. The first author, who conducted the interviews, asked the participants of the study to describe what places, they met with people, the character of these meetings as well as the social relationships they established. A certified interpreter was engaged for two interviews, performing simultaneous translation. The participants in the study chose the place for the interview. The first author conducted seven interviews in the older person’s home and three in another location, such as a conference room in a community Centre. The interviews ranged from 60 to 90 minutes, were audio-recorded, and transcribed verbatim. All participants signed an informed consent form prior to the interviews. Ethical approval for the study was received from the Regional Ethical Review Board in Gothenburg (reference #821–11, #001–12). The analysis of the interviews followed the guidelines in Malterud’s systematic text condensation (2012). Discussions between the authors took place at every step of the analysis. Initially, the audio material and the transcripts were read through and listened to several times to obtain an overall impression of the collected data, going from chaos to preliminary themes. Then, meaning units were distinguished through the text that met the aim of the study and were sorted into four code groups. The meaning of each code group was condensed and written about as if it were a story told by the participants. Next, the content of each code group was synthesized and given a name to provide a new understanding of the studied phenomenon [21]. The results were validated by checking the transcribed interviews against the synthesized code groups (presented in the results) and by regular discussions among the authors throughout the analysis.

## Results

The analysis resulted in four code groups with nine subcategories: Everyday places become meeting places; Organized meeting places support social contacts; Meeting places as identity markers and Barriers to accessing meeting places. The four groups entailed nine subcategories (see Table 1).

**Table 1:** Older people’s perceptions of places to meet.

Everyday places become meeting places	Organized meeting places support social contacts			Meeting places as identity markers		Barriers to accessing. meeting places.		
Community and trust	Member-ship groups	Voluntary groups	Municipality groups	Choice identity	Avoidance identity	Perceptions of not belonging	A strained financial situation.	Limited Swedish proficiency
Example: a staircase in an apartment block, at a bus stop or on a bus	Example: Condominium association	Example: the bridge club	Example: Senior centers	Example: Senior centers	Example: various associations and fellowships	Example: former workplace, a retirement club		

### Everyday places become meeting places

#### Community and trust

Participants made new connections. It emerged that everyday meeting places, whose purpose was not primarily to provide a social meeting place, did have such a function. Social meetings could occur on a staircase in an apartment block, at a bus stop or on a bus, contributing to feelings of community and trust. Recurrent meetings between individuals at such places could gradually evolve into closer relationships where support was exchanged.

"I got to know a person from the same neighborhood at the bus stop. She has lived here for forty-three years: we became friends … a few days ago, I had to go to the medical center as she no longer knows where it is. She cannot find her way there on her own."

The everyday places served as an intermediate step to deepen the relationships that could continue outside this context. Furthermore, the social interaction that occurred at these meeting places also appeared to have value in itself.

"These days, I take the public shuttle service where I socialize with other people; there are many people I can have a good laugh with…we talk to each other, and it is delightful."

### Organized meeting places support social contacts

#### Membership groups

Some meeting places required membership to gain access and entailed a shared responsibility between the members. In addition to the "contract" in terms of membership, social support could be given at these meeting places that exceeded the required duties. One example of this was the condominium association. The joint responsibilities for the premises, gardening and other things that had to be done created a meeting place that allowed an exchange of support between the members that went beyond their duties, such as jointly celebrating birthdays or cooking for someone who had recently arrived home from the hospital:

"The neighbor living across from me [member of the condominium association] brought me food when I came home from the hospital when I could not cook for myself. They thought that cooked food would come in handy."

#### Voluntary groups

Also, a bridge club was found to provide a basis for the creation and maintenance of social contact. The responsibility toward each other was not mandatory in the same way as appeared to be the case in the condominium association; the tasks performed here were more voluntary. Everyone knew each other, but not on a deeper level, as became apparent in the interviews with the participants. However, an awareness of each other could emerge in such groups; for example, someone lonely could be given extra attention. Additionally, a gradual establishment of social contact was evident; weekly greetings could eventually lead to an exchange of support.

“I met an uncommunicative man at the bridge club I usually go to. He could have been more communicative; he did not talk to anyone. I told my wife I would help him to open up… One day, he came up to me … He said: "I trust you so that I will give you the keys to my apartment.…will you please check my mail while I am gone?" I said, "OK, I will." Then he said, "So, is there anything I can do for you?".

#### Municipality groups

Meeting places organized by the municipality, such as senior centers, also served the purpose of mutuality for the participants. An example of such a meeting place was the support group for carers.

"I feel free [when attending the support group for carers], and I do not think about my problems so much; I become happier, too.…they mean a great deal to me, so I assume that I mean a great deal to them too."

### Meeting places as identity markers

#### Choice identity

Different meeting places also reflect the personal preferences of the interviewees. This was shown in the choice or avoidance of certain meeting places. The participants chose meeting places they identified themselves with. Some interviewees were, for example, involved in various associations and fellowships, viewing themselves as "fellowship people". Others regarded themselves as "dog people", for whom the daily walks with the dog were a meeting place for new acquaintances. Furthermore, they also described exploring their identity in different meeting places.

"I am not in a retirement club. I was for a while, but I did not think I got anything from it, so …I thought it was better to walk the dog… that gave me the opportunity to meet other people."

#### Avoidance identity

Some meeting places were avoided because they reminded me of lost identities and no longer belonging. It could be the former workplace, a retirement club or somewhere else. "When I quit my job, I was anxious about leaving it; later, when I met my former colleagues, they said they missed me. I said I miss you too, but I can do nothing about it… I have not returned once since I retired due to anxiety over the separation I experienced." For others, feelings of affiliation emerged in experiences of how they maintained contact with old colleagues. By meeting former colleagues once a month at a local pub, participants could maintain a sense of identity-related to the former workplace. These meetings rekindled the feeling of being a part of the past community. The affiliation experience could also emerge when meeting former classmates from the country of origin at class reunions. The home country was a place that made them reflect upon how their own life had turned out compared to those who had stayed in their home country.

"I now keep in touch with my former high school classmates in my country of birth… In these meetings, we get insight into what has happened and how we have aged. It is interesting to see how they are doing and what they do in their spare time. For some interviewees, their homes represented the central venue and meeting place in their lives, which was used for maintaining contact with family and friends. Thus, more public meeting places such as associations and fellowships did not appeal to these participants."

#### Barriers to accessing meeting places

Some aspects of meeting places were described as obstacles to participation. Barriers were perceptions of not belonging, strained financial situations, and limited Swedish proficiency.

#### Perceptions of not belonging

Perceptions of not belonging as a barrier mainly occurred as perceptions of feeling too old and functionally limited or too young and healthy to go to. Only a few participants associated barriers to participation with their previous migration.

“You stand on a bridge with half the body on one side and half the body on the other. Mm, ok, in two worlds all the time, I can tell you… If I’m not mistaken, almost everyone feels that way.”

The interviewees reported how they associated certain meeting places with decline and frailty for older adults, which made them reluctant to go there. However, some interviewees challenged these notions and attended meeting places that others associated with negative aspects of ageing.

#### A strained financial situation

A strained financial situation could also be experienced as an obstacle to participation in particular meeting places. Being unable to travel due to friends or not having the necessary financial means could lead to difficulties in maintaining or establishing social contacts.

#### It is hard to find new friends my age willing to travel

I don’t know; maybe they are afraid. For the most part, due to economic problems.

“Women born in the forties had low-paid jobs and had no money to spend except on the most essentials."

#### Limited Swedish proficiency

Language was also found to be a barrier to participation in meeting places where most participants spoke Swedish. At such meeting places, participation was therefore experienced as more difficult. Where the workplace no longer represented a possible social contact arena, several interviewees mentioned its importance for learning the language. Others stressed how the lack of a workplace as a meeting place affected their present possibilities to establish contacts with Swedish-speaking people in later life and to participate in Swedish-speaking meeting places." I would like to have the opportunity to meet someone who is native Swedish.

"I want to be able to talk to them. I am now of an age at which I do not work, and I will not work again since I am retired. The workplace creates a network of people to talk to, providing an opportunity to practice Swedish. "

## Discussion

The findings show that the participants attended various meeting places to establish and maintain social contacts. Previous literature on older migrants’ participation in meeting places, in the Swedish context, has focused on meeting places targeting older people from a specific country ([13–15]). However, the results of this study demonstrate the significance of meeting places that do not specifically target older people in general or older people from a specific country. The results demonstrate how everyday places in the neighborhood, which were not primarily meant to be meeting places, were used to create and uphold social contacts and thus promoted social inclusion. These results align with previous findings [22]. Research focusing on meeting places for older adults has often studied the significance of senior centers (Dal Santo, 2009). In addition, the participants told how they formed social relationships at different associations or clubs created for social purposes. These results demonstrate that the participants had access to social contexts where they established social contacts. Nevertheless, previous studies show that older migrants in Sweden are less likely to participate socially than Swedish-born older adults [23]. Further research is therefore needed to investigate in more depth how older migrants’ social relationships can be promoted on an overall level; the participants’ experiences of the significance of the meeting places and the social relationships they brought could be understood as contributing to a sense of belonging [23]. Meeting with and establishing bonds with people in everyday places in the neighborhood contributed to experiences of community and trust. Other places where they had more actively engaged themselves, such as the condominium association, were also felt to provide mutuality. However, every day and formal meeting places were used to establish and develop relationships that could result in an exchange of practical and emotional support. These results align with some previous Swedish studies that describe the local community as a context for care and support ([24, 25]).

Furthermore, the participants’ self-identification and identification with others emerged as gate openers and gatekeepers in accessing various meeting places. Perceptions of not belonging to certain meeting places could form barriers preventing participation in some meeting places. As shown before, self-identification and identification with others could contribute to a sense of belonging [26]. These perceptions mainly occurred as an idea of being too old or young to visit certain meeting places. Such attitudes can be interpreted as ageism, i.e., revealing internalized negative attitudes towards old age and ageing, restricting older people’s thoughts and actions [27, 28]. Furthermore, they reveal how stereotypical images of ageing may harm the attractiveness of organized meeting points geared to older people.

In line with previous research, we argue that senior centers need to adapt to the diverse and changing needs of the older population ([10, 11]). Strained financial situations also hinder participating in comparatively expensive activities like travelling. Previous findings highlight how factors such as previous work history and marital status interact with financial constraints in old age, especially for women [29, 30].In addition, some of the participants pointed to language as a barrier to participating in meeting places where the majority spoke Swedish. These experiences can be interpreted as different degrees of social inclusion, gaining access but not participation [31], which is in line with previous literature indicating that restricted language proficiency in the new country constitutes a barrier for older migrants in making social contacts [4]. Although all the participants were retired, and thus, the workplace no longer offered a possible meeting place for social contacts; having a job after migration seemed to be of great significance for whether a person had learned Swedish. These results highlight the significance of previous integration in the labour market for social participation in old age. While barriers such as ageism may not be unique to older migrants, financial constraints and the language barrier might be. Previous literature demonstrates that migrants in Sweden are more frequently exposed to social exclusion, such as chronic unemployment and economic problems [2, 32]. The participants’ perceived obstacles in accessing meeting places due to the language barrier and financial constraints may also be understood as differences in migrants’ and Swedish-born older adults’ capabilities [33]. Thus, even though older people may have access to the same meeting places, they might not have the same possibility. Finally, the results show that older migrants’ life conditions should be considered in terms of how experiences and historical events affect the person’s life story, considering barriers to access.

### Meeting places exist on both the individual and the structural levels

Finally, in qualitative research, external validation concerns whether or not the results can be applied in other settings ([21, 34]). The sampling of the study has been done based on previous research showing that ill health is more prevalent among older migrants, as is a lower likelihood of social participation among older migrants compared to Swedish-born older adults, thus warranting the aim and the research question of the current study. The results are thus viewed as applicable in a particular context with implications for other contexts [21]. The participants in this study have all chosen to participate in a health-promotion study. Consequently, the participants might be considered a “spirited group of pensioners” not representative of the larger heterogeneous group of older migrants. Even though the sample in this study reflects some heterogeneity regarding migration background, gender, and age at migration, the results can still be seen as an essential contribution to this relatively unexplored research field.

### Implications for policy and practice

This study provides valuable insights, especially regarding gerontological social work promoting social participation in society for all older adults and meeting places where they can contribute to or limit such opportunities for older migrants. The results of this study point to the importance of creating environments, so-called everyday meeting places, which can naturally enable meetings between older people. This is particularly important for people who suffer financial constraints. Creating everyday meeting places is of fundamental importance in health promotion for older people in Sweden, especially those with financial vulnerability who are also at greater risk of poor health. Since everyday places that were not designed or intended as meeting places proved to be of great importance, we argue that there is a need to move toward a more comprehensive definition of meeting places for older adults. Our findings on older migrants’ perceptions of not belonging to certain meeting places should urge policymakers and practitioners working with older people to find ways of promoting social participation that reflect the increased heterogeneity of the older population. Moreover, as our study reveals different barriers to older migrants’ social participation in some meeting places, we need to analyze further how social exclusion and inclusion processes are used.
